# Rationale for the Turin Conference/Congress 2012

**DOI:** 10.1186/2049-6958-7-1

**Published:** 2012-06-11

**Authors:** Claudio F Donner, Carlo Gulotta

**Affiliations:** 1 Chairman, 7th, Pulmonary Advances, Turin, Italy; 2 Co-Chairman, 7th, Pulmonary Advances, Turin, Italy

## 

In 1987 the first International Conference on the Management of Chronic Respiratory Failure was held in Veruno (Italy). Twenty five years and six Conferences later much has changed, not only in regard to the organization of the Conference itself (e.g., now it is held every three years instead of five as originally), but also the problems arising from chronic respiratory diseases (CRDs) have changed. Today, according to World Health Organisation’s (WHO) estimates, some 300 million people worldwide suffer from asthma and 80 million people are affected by moderate to severe COPD. These diseases are among the most important causes of death in our society and their burden is constantly increasing. Over the last 20 years effective technical and pharmacological aids have been added to the tool-box of the chest physician, improving the life of patients both in terms of expectancy and quality. Obviously, this development of chest medicine has also had important repercussions on health resources consumption and currently there is a widespread debate on how to cope with the growing burden. The International Conference on “Management and Rehabilitation of Chronic Respiratory Failure” - now in its 7^th^ edition - is recognized as a key international scientific event in this specific field.

The 2012 “Pulmonary Advances”, which will be held in Turin, will deal with new developments in respiratory medicine as well as with their cost-effectiveness and the best way to deliver them, with particular focus on the possibilities of integration between specialists and primary care medicine. Two major scientific events will run in parallel: the 7^th^ International Conference and the 3^rd^ National Congress of AIMAR (Interdisciplinary Scientific Association for Research in Lung Disease). The novel idea underpinning AIMAR is that management of respiratory diseases should not be solely in the hands of the pneumologists but should be multidisciplinary. This approach goes beyond the purely ‘organ specialist’ outlook prevalent in clinical practice, integrating the diverse professional figures (including non-medical health professionals) to promote, through continuing professional education, the development of joint clinical controlled studies, epidemiological surveillance and public health education in order to lay the foundations for an optimal management of respiratory diseases. The Conference/Congress of Turin 2012 will thus have a clear interdisciplinary character, offering the maximum in terms of quality, up-to-date information in the fight against and prevention of CRDs. This is in harmony with the scope of GARD (Global Alliance against Chronic Respiratory Diseases), a WHO-based voluntary alliance of national and international organizations aimed at improving the respiratory health of the general population by fighting the current problem of underdiagnosis and undertreatment of CRDs. The benefit of this is very evident to professionals, health planners and decision makers, but what benefit is there for the patient and general public? There is nothing but to gain from a greater flow of information between the various specialists and a more integrated mode of clinical practice. Patient management will no longer be - as it is often today - the fruit of improvised collaboration, but it will be based on a global, comprehensive model of care that coordinates the up-to-date expertise of diverse specialists. Moreover, assuring patients a homogeneous protocol of diagnosis and treatment irrespective of what specialist branch they consult for a respiratory problem will give them the added security of knowing they are cared for by professionals who have a united vision of their case. In short, AIMAR represents a great alliance between physicians, health professionals and patients for the lungs, which means a great alliance for the benefit of all citizens. Our wish is that the commitment manifested by the Scientific Committees and Organizing Committee - in their respective areas of competence - combined with the work of the almost 200 speakers and a fruitful exchange of opinions among participants will enable us to realize that particular “mix” that will make this a unique scientific event.

